# A prognostic systemic inflammation score (SIS) in patients with advanced intrahepatic cholangiocarcinoma

**DOI:** 10.1007/s00432-022-04424-0

**Published:** 2022-11-05

**Authors:** M. Maßmann, J. Treckmann, P. Markus, B. Schumacher, D. Albers, S. Ting, B. Mende, J. Roehrle, I. Virchow, V. Rosery, K. Laue, G. Zaun, K. Kostbade, M. Pogorzelski, K. W. Schmid, H. Baba, J. T. Siveke, A. Paul, H. U. Schildhaus, M. Schuler, M. Wiesweg, S. Kasper

**Affiliations:** 1 West German Cancer Center, Department of Medical Oncology, University Hospital Essen, Hufelandstr. 55, 45147 Essen, Germany; 2 West German Cancer Center, Department of General, Visceral and Transplant Surgery, University Hospital Essen, Essen, Germany; 3 Department of General Surgery and Traumatology, Elisabeth Hospital Essen, Essen, Germany; 4 Department of Gastroenterology, Elisabeth Hospital Essen, Essen, Germany; 5 West German Cancer Center, Institute of Pathology Essen, University Hospital Essen, Essen, Germany; 6 Central Pharmacy, University Hospital Essen, Essen, Germany; 7 German Cancer Consortium (DKTK), Partner Site University Hospital Essen, Essen, Germany; 8 Bridge Institute of Experimental Tumor Therapy, West German Cancer Center, University Hospital Essen, Essen, Germany; 9 Medical Faculty, University Duisburg-Essen, Essen, Germany; 10 Division of Solid Tumor Translational Oncology, German Cancer Consortium (DKTK, Partner Site Essen) and German Cancer Research Center, DKFZ, Heidelberg, Germany

**Keywords:** Intrahepatic cholangiocellular carcinoma, Chemotherapy, Advanced disease, Inflammation, Systemic-inflammatory response parameters, Prognosis

## Abstract

**Purpose:**

Systemic-inflammatory response parameters (SIR) are known prognostic markers in different tumour entities, but have not been evaluated in patients with iCCA treated with systemic chemotherapy. Therefore, we evaluated the impact of different SIR markers on the clinical course of patients with advanced iCCA treated at our center.

**Methods:**

SIR markers were retrospectively evaluated in 219 patients with iCCA at the West-German-Cancer-Center Essen from 2014 to 2019. Markers included neutrophil/lymphocyte ratio (NLR), lymphocyte/monocyte ratio (LMR), CRP, and the modified Glasgow-Prognostic-Score (mGPS), which were correlated with clinico-pathological findings, response to chemotherapy (ORR), progression-free (PFS) and overall survival (OS) using Kaplan–Meier analyses, and Cox proportional models.

**Results:**

Median overall survival (OS) of the entire cohort was 14.8 months (95% CI 11.2–24.4). Median disease-free survival (DFS) in 81 patients undergoing resection was 12.3 months (95% CI 9.7–23.1). The median OS from start of palliative CTX (OS_pall_) was 10.9 months (95% 9.4–14.6). A combined Systemic Inflammatory Score (SIS) comprising all evaluated SIR markers correlated significantly with ORR, PFS, and OS_pall_. Patients with a high SIS (≥ 2) vs. SIS 0 had a significantly inferior OS_pall_ (HR 8.7 95% CI 3.71–20.38, p < 0.001). Multivariate analysis including known prognostic markers (ECOG, CA19-9, LDH, and N- and M-status) identified the SIS as an independent prognostic factor.

**Conclusions:**

Inflammatory markers associate with inferior survival outcomes in patients with iCCA. A simple SIS may guide treatment decisions in patients treated with systemic chemotherapy.

**Supplementary Information:**

The online version contains supplementary material available at 10.1007/s00432-022-04424-0.

## Introduction

Biliary tract cancer (BTC) is a rare malignant disease with an incidence of 1/100.000 per year in Western countries (Massarweh and El-Serag 2017). BTC consists of three different sub-entities with distinct molecular alterations: intrahepatic cholangiocarcinoma (iCC), extrahepatic cholangiocarcinoma (eCCA), and gall bladder cancer (GC). The iCCA accounts for up to 20% of newly diagnosed liver cancers with increasing incidence and mortality in recent years (Massarweh and El-Serag 2017; RKI 2019). The only curative option of this disease is surgical R0-Resection. Based on the data of the BILCAP trial, which showed a prolongation of overall- and recurrence-free survival in the per protocol population, an adjuvant chemotherapy with capecitabine after curative resection is recommended (Primrose et al. 2019). However, even after curative resection and adjuvant chemotherapy, most patients have a relapse with a median disease-free survival (DFS) of 12–36 months and 5-year survival rates of only 20–40% (Blechacz 2017; Rizvi et al. 2018). Unfortunately, most patients are diagnosed in advanced, non-resectable or metastatic stage and are treated with systemic palliative chemotherapy. Gemcitabine and Cisplatin remain the standard of care based on the results of the ABC-02 trial (Lamarca et al. 2014). The recently published TOPAZ-1 trial showed a superiority of the immune-checkpoint inhibitor durvalumab in combination with gemcitabine and cisplatin compared to gemcitabine and cisplatin alone, and this combination could be considered as the new first-line standard of care in patients with advanced CCA (Oh et al. 2022). After failure of first-line chemotherapy, fluoropyrimidine-based regimens have shown activity in clinical trials (Valle et al. 2010; Yoo et al. 2021). Especially in iCCA, distinct molecular alterations amenable to targeted treatment, such as rearrangements of the fibroblast growth factor receptor 2 (FGFR) or mutations of the isocitrate dehydrogenase 1 (IDH1), are detected in up to 30% of patients (Abou-Alfa et al. 2020). Targeted drugs have become a new standard for these patients after failure of gemcitabine-based first-line therapy (Rizzo et al. 2020). The FGFR inhibitor pemigatinib has been approved by FDA and EMA for patients with BTC harbouring FGFR2 rearrangements and ivosidenib has been approved by the FDA for patients with IDH1-mutant BTC (Abou-Alfa et al. 2020). For most patients though, cytotoxic chemotherapy remains the mainstay of therapy with a median overall survival (mOS) of only 11–13 months. Furthermore, a substantial number of patients do not benefit from combination chemotherapy regimens, or intensive chemotherapy protocols cannot be administered due to comorbidities (Lamarca et al. 2014).

Main risk factors for the development of iCCA are chronic inflammatory diseases, such as primary sclerosing cholangitis (PSC), and Hepatitis B or C (Massarweh and El-Serag 2017). Chronic inflammation is triggered by the release of cytokines, followed by cell death and proliferation of biliary and hepatic progenitor cells (Palmer and Patel 2012; Razumilava and Gores 2014). In addition, intra-tumoural inflammation triggers tumour progression through DNA damage and activation of intracellular signalling pathways (Hanahan and Weinberg 2011).

Chronic inflammation is usually associated with an activation of the systemic immune system. This activation can be easily assessed by blood-based parameters, such as the C-reactive protein (CRP) or subsets of white blood cells.

Against this background, we aimed to investigate the impact of markers of systemic inflammation on the clinical outcome in patients with iCCA treated with systemic chemotherapy. For this, we retrospectively correlated a predefined systemic inflammation score (SIS) based on the following systemic inflammatory response parameters (SIR): the modified Glasgow Prognostic Score (mGPS), neutrophil–lymphocyte ratio (NLR), lymphocyte–monocyte ratio (LMR), and CRP with the response to systemic chemotherapy, progression-free survival, and overall survival in a cohort of patients with iCCA (Markus et al. 2021; McNamara et al. 2014; Okano et al. 2018; Proctor et al. 2011; Schweitzer et al. 2017). In previous studies, these markers have been identified as strong prognostic factor in other malignancies such as pancreatic cancer (Markus et al. 2021).

## Methods

### Study design

Patients with histologically confirmed iCCA treated between 2014 and 2019 at the West German Cancer Centre, Essen, Germany were retrospectively identified. Patients with resection in curative intent and patients with advanced, non-resectable disease who received systemic chemotherapy were enrolled into this retrospective study. Baseline characteristics and treatment history were extracted from the electronic health record (EHR). Clinical data were anonymized for analysis. The following SIR parameter were analysed: NLR, LMR, CRP, mGPS—a composite marker of high CRP-levels (> 1 mg/dl) and low albumin levels (< 3.5 g/dl) (Proctor et al. 2011). We chose the following cut-off values to define a systemic inflammation score (SIS): NLR ≥ 5, LMR ≤ 2.8, CRP ≥ 5 mg/dl, and mGPS ≥ 1. For each positive marker, one point was allocated and patients were separated into three groups with low, intermediate, and high risk. This prognostic SIS was previously identified and validated by our group in an independent cohort of patients with advanced pancreatic cancer (Markus et al. 2021).

The study was approved by the Ethics Committee of the Medical Faculty of the University Duisburg-Essen (Project No. 15-6497).

### Assessments

Laboratory values were assessed at diagnosis and before the start of palliative chemotherapy. Tumour response was routinely assessed by computed tomography (CT) or magnetic resonance imaging (MRI) before start of palliative chemotherapy and subsequently every 8–12 weeks according to institutional guidelines. Treatment response was evaluated according to the Response evaluation criteria in solid tumours 1.1 (RECIST). Objective Response Rate (ORR) was defined as the percentage of patients with complete or partial response and Disease Control Rate (DCR) was defined as the percentage of patients with complete response, partial response, or stable disease in at least on follow up. Patients were eligible for response evaluation if one baseline radiological examination and one follow-up were available. Overall survival (OS) was defined as time of diagnosis until death of any cause. OS_pall_ was defined as the start from palliative treatment until death of any cause. OS_cur_ was defined as the start of curative treatment until death of any cause. Patients were censored at the time of last follow-up. Disease-free survival was defined as the time from curative resection until relapse or death. Progression-free survival (PFS) was defined as the time from the beginning of systemic therapy to the date of radiological progression according to RECIST 1.1, or death.

### Statistics

Statistical analyses were performed using R 3.6 and the tidyverse (R Core Team 2018; Wickham et al. 2019). Plots, survival analyses (log-rank tests, Cox proportional hazard model), and Kaplan–Meier plots were generated using the packages ggplot2 3.3, survival 3.2, survminer 0.4.9 and survivalAnalysis 0.2 (Kassambara et al. 2017; Therneau 2022; Wickham 2016). Median follow-up was assessed by the reverse Kaplan–Meier method.

## Results

### Patients

A total of 219 patients were enrolled into this retrospective study (Suppl. Figure 3). Baseline patient characteristics are presented in Table 1. The median age at diagnosis was 64.9 years (range 25.7–87.8 years). In total, 81 patients (37%) had a curatively intended tumour resection, 54 (66.7%) of these had a relapse. The remaining 102 patients (47%) received palliative systemic treatment, best supportive care, or deceased shortly after initial diagnosis prior to initiation of any systemic therapy due to advanced, non-resectable disease or metastases (30.6%). For 36 patients, no follow-up and no further information were available. At time of diagnosis, 61.5% of the patients with available data had elevated CA19-9 levels (median 69.9 U/ml; normal (< 37–118,958 U/ml) and 63.8% of patients had an LDH above the upper limit of normal (> 247 U/l) (Table 2). The median follow-up time was 48 months (95% CI 40.9–54.7). At time of data cut-off, 159 (72.6%) patients were deceased, and 35 (16%) patients were lost to follow up.

**Table 1:** Baseline characteristics

	*N*	%
Sex		
Male	111	50.6
Female	108	49.4
Median Age at Dx (range)	64.9 (25.7–87)	
Age		
< 70 years	143	65.5
> 70 years	76	34.5
ECOG		
0	29	13.2
1	22	10
> = 2	24	11
n.d	144	65.8
T-status		
1	48	21.9
2	85	38.8
3	46	21
4	26	11.9
n.d	14	6.4
N-status		
0	125	57.1
+ /1/2	84	38.4
n.d	10	4.6
M-status		
M0	152	69.4
M1	67	30.6
n.d	0	0
Median follow-up in months (95% CI)	48.0 (40.9–54.7)	
Mean tumour size in mm (range)	87.9 (0–241.16)	

*Dx* diagnose, *ECOG* Eastern cooperative oncology group

**Table 2:** Prognostic factors

Prognostic factors	Median (range)	Cut-off	N > cut-off	N < cut-off
NLR	4.41 (1.09–48.13)	≥ 5	38	53
LMR	2.15 (0.65–5.12)	≤ 2.8	17	73
CRP	2.1 (0–67)	≥ 5 mg/dl	37	84
m-GPS				
0	35	≥ 1 point	47	35
1	31			
2	16			
CA 19-9	69.6 (normal-118958)	> 37 U/ml	64 (29.2%)	40 (18.3%)
LDH	279.5 (131–3607)	ULN > 240 U/ml	74 (33.8%)	42 (19.2%)

*NLR* neutrophil–lymphocyte ratio, *LMR* lymphocyte–monocyte ratio, *CRP* C-reactive protein, *m-GPS* modified Glasgow prognostic score, *CEA* carcinoembryonic antigen, *CA19-9* carbohydrate antigen, *LDH* lactate dehydrogenase, *ULN* upper limit of normal

### Survival

**Fig. 1:**
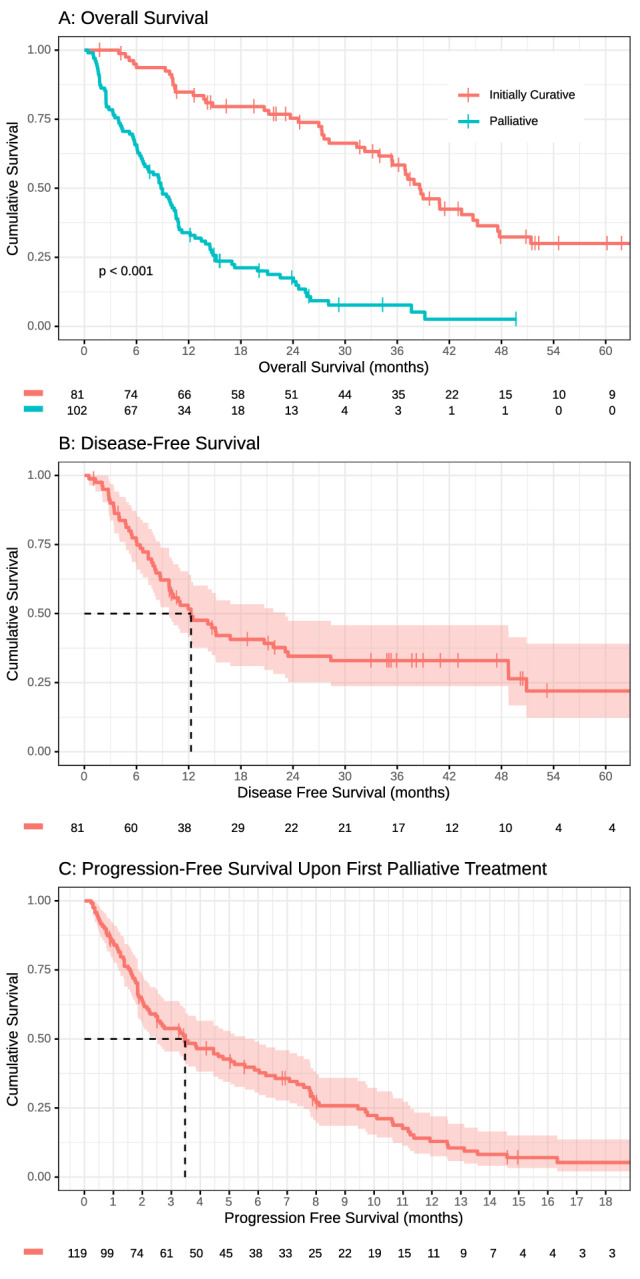
**A** Overall survival, **B** disease-free survival, and **C** progression-free survival of the patient cohort

Overall survival of the entire study population was 14.8 months (95% CI 11.2–24.4 months). DFS of the 81 patients who were resected in curative intent was 12.3 months (95% CI 9.7–23.1). Median OS of resected patients was 38.6 months (95% CI 35.4–47.6 months) (Fig. 1A, B). The median OS for patients, which only received palliative treatment upfront (*n* = 102) was 8.8 months (95% CI 7.0–10.6). The OS_pall_ of the 150 patients who either had a disease recurrence or metastases was 10.8 months (95% CI 9.4–14.6) (Suppl. Figure 1).

### Efficacy of palliative chemotherapy

**Fig. 2:**
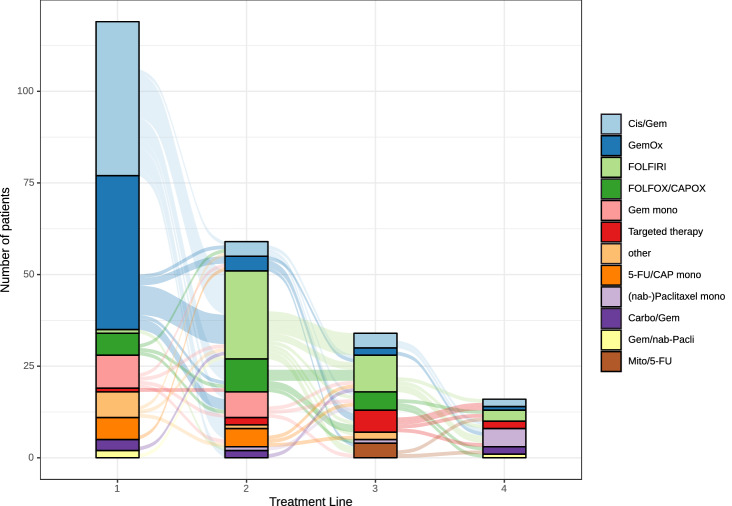
Chemotherapy regimen and conversion in first, second, third, and fourth treatment line. *Cis/Gem* Cisplatin and Gemcitabin, *GemOx* gemcitabin and oxaliplatin, *FOLFIRI* folinic acid, irinotecan and fluorouracil, *FOLFOX* folinic acid, fluorouracil, oxaliplatin, *CAPOX* capecitabin and oxaliplatin, *Carbo/Gem* Carboplatin and Gemcitabin, *Mito/ 5-FU* mitomycin and fluorouracil

In total, 119 patients (54.3%) received systemic first-line chemotherapy either upfront or after disease recurrence. Of these, 59 patients (49.6%) received second-line therapy and 50 patients (28.6%) received three lines of palliative chemotherapy. The majority of patients were treated in first line with cisplatin and gemcitabine (35.3%) or oxaliplatin and gemcitabine (35.3%) (Fig. 2). After failure of first-line gemcitabine-based therapy, most patients received combination therapies with fluoropyrimidines and irinotecan or oxaliplatin (FOLFIRI) or FOLFOX (Suppl. Table 1, Fig. 2).

**Fig. 3:**
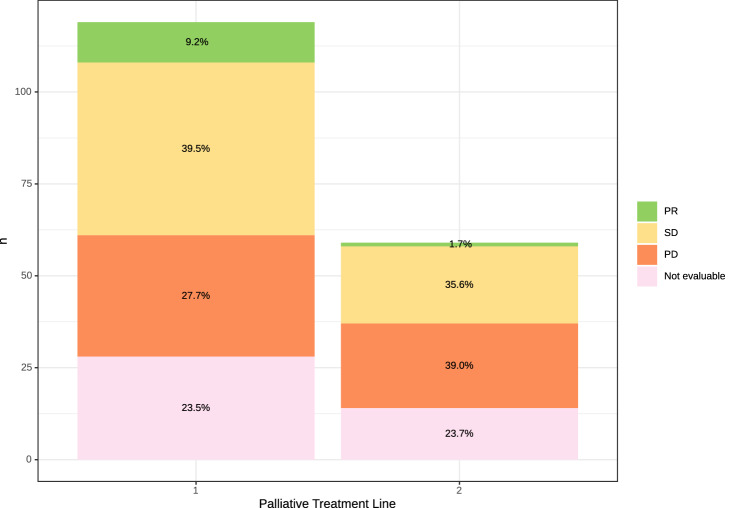
Best objective response rate to palliative treatment. *PR* partial response, *SD* stable disease, *PD* progressive disease

In total, 91 patients (77.3% of 119 treated with palliative systemic therapy) were evaluable for radiological response according to RECIST 1.1; 28 patients (23.5%) had no baseline or follow-up scans and were not evaluable. The ORR to first-line therapy was 9.2% and the DCR was 48.7%; 27.7% of patients had progressive disease in the first radiologic re-evaluation (Fig. 3, Suppl. Table 2). The median PFS upon first-line chemotherapy was 3.5 months (95% CI 2.5–5.5 months) (Fig. 1C). For second-line systemic therapy, ORR was only 1.7%, and DCR was 37.3% (Fig. 3). The median PFS-2 upon second-line chemotherapy was 2.6 months (95% CI 1.9–3.9 months).

### Systemic-inflammatory response parameters (SIR) as prognostic biomarkers

**Fig. 4:**
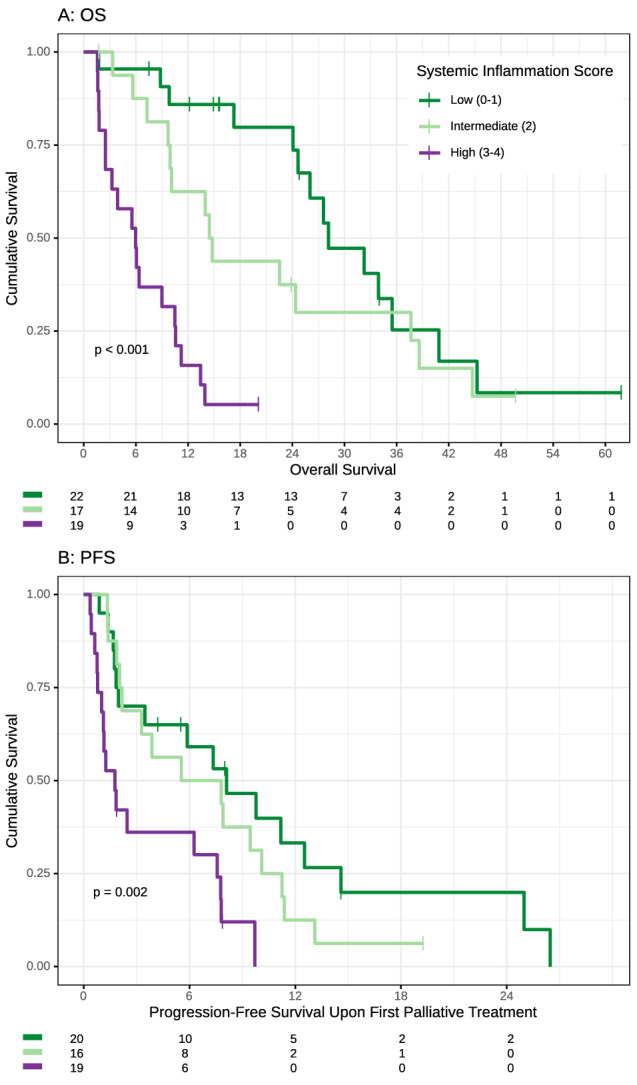
**A** Overall survival of patients according to the systemic inflammation score, patients were grouped according to their score. **B** Progression-free survival of patients according to the systemic inflammation score, patients were grouped according to their score

**Fig. 5:**
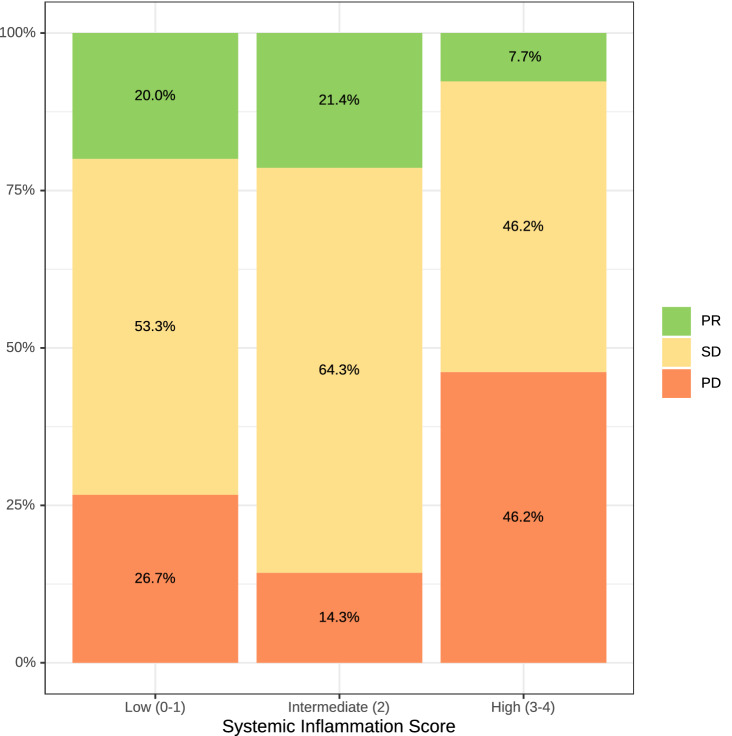
Best objective response rates to palliative treatment Line 1 and 2 in patients according to the Systemic Inflammation Score. *PR* partial response, *SD* stable disease, *PD* progressive disease

The SIR markers were assessed in patients at treatment initiation. For the NLR and LMR, data of 91 and 80 patients, and for the mGPS and CRP, data from 82 and 121 patients were available. In our cohort, 38 patients (41.76%) had an elevated NLR, 73 patients (91.25%) had a reduced LMR, 37 patients (45.12%) had an elevated CRP, and 47 patients (38.84%) had an mGPS above the previously identified and validated cut-off values (Table 2). Based on previous publications (Markus et al. 2021), for each positive SIR marker, one point was allocated and the patients were sorted into three groups of presumably increasing risk: low (0–1), intermediate (2), and high systemic inflammation score (SIS) (3–4). Of 58 patients with available SIS, 22, 17, and 19 patients were allocated to the groups low, intermediate, and high SIS (Fig. 4). Median OS of patients in the low SIS group was 28.2 months (95%CI 24.7–NA months), in the intermediate SIS group 14.6 months (95% CI 9.9–44.7 months) and in the high SIS group only 6.0 months (95% CI 3.2–11.2 months) (Suppl. Table 3). There was no statistically significant difference in the OS between patients with a low and intermediate SIS (HR 1.64, 95% CI 0.77–3.47, *p* < 0.196). In contrast, patients with high SIS had a significant reduced OS compared to patients with a low SIS (HR 8.7, 95% CI 3.71–20.38, *p* < 0.001) (Suppl. Table 3). Next, we correlated the SIS with the response to systemic first-line therapy (Fig. 5). Patients with a low SIS had a numerically higher chance to achieve a disease control (73.3%) compared to patients with a high SIS (53.9%). In line, 46.2% of patients with high SIS, had progressive disease in the first radiological examination after start of palliative chemotherapy. Furthermore, median PFS was significant higher in patients with lower SIS (Fig. 4). Patients with a low SIS had a median PFS of 8.1 months, whereas patients with a high SIS had only a median PFS of 1.8 months (HR 3.56, 95% CI 1.65–7.65 *p* 0.001) (Suppl. Table 4).

### Multivariate analyses

**Fig. 6:**
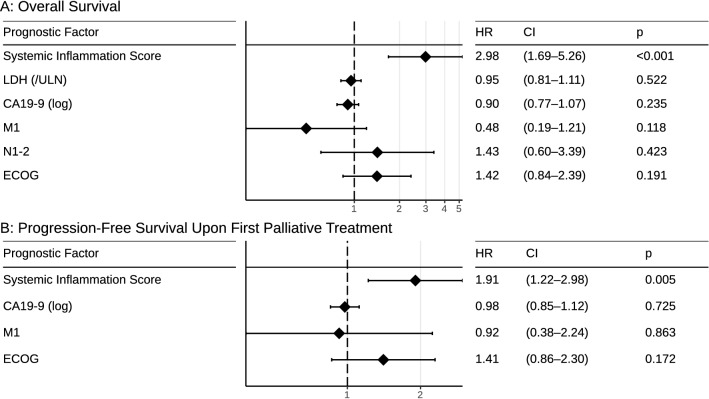
Multivariate analysis of the prognostic factors. Factors were only included if they were statistically significant in the univariate analysis. The SIS was entered as a continuous variable (range 0–4). *LDH* lactate dehydrogenase, *CA19-9* carbohydrate antigen, *ECOG* Eastern cooperative oncology group

To verify the independence of our new proposed SIS for OS and PFS, we investigated this score in a multivariate analysis with known prognostic factors including ECOG, CA 19-9, LDH, and N- and M-status (Fig. 6). For OS and PFS, only the SIS was an independent prognostic factor (OS HR 2.98; PFS HR 1.91 *p* < 0.001 SIS) in this analysis (Fig. 6).

## Discussion

The systemic treatment of patients with unresectable or recurrent BTC remains challenging. Despite some progress in the last years with the implementation of effective second-line cytotoxic and molecular targeted therapies, the prognosis is still poor with median OS times of only one year. Recently, the addition of the PD-L1 checkpoint inhibitor durvalumab to gemcitabine and cisplatin showed a statistically significant prolongation of median OS compared to gemcitabine/cisplatin alone in the randomized phase III TOPAZ-1 study (Oh et al., 2022). The clinical benefit was only moderate with a prolongation of median OS from 11.5 to 12.8 months (HR 0.8, 95% CI 0.66–0.97). However, a subgroup of patients in this trial had a sustained response to this therapy, but prognostic or predictive markers to identify this subgroup are missing. After failure of first-line therapy with gemcitabine/cisplatin, two phase III trials showed the efficacy of second-line therapy. In the ABC-06 trial, FOLFOX was superior to active symptom control, and in the NIFTY trial, liposomal irinotecan/fluorouracil was superior to fluorouracil and leucovorin after failure of gemcitabine-based first-line therapy (Lamarca et al. 2021; Yoo et al. 2021). Unfortunately, the superiority of liposomal irinotecan to fluorouracil and leucovorin versus fluoropyrimidin/leucovorin alone could not be confirmed in the prospective NALIRICC study in a western patient population. Thus, fluoropyrimidin/leucovorin could be an alternative, less-toxic second-line therapy (Vogel et al. 2022). However, only patients with good performance status and without significant comorbidities are usually enrolled into such randomized clinical trials. In clinical routine, patients with BTC often show impaired liver function or recurrent cholangitis and the administration of cytotoxic combination therapies remains challenging. In addition, a significant number of patients do not benefit from intensive cytotoxic chemotherapy but suffer from side effects with a deterioration in quality of life. Predictive and prognostic biomarkers are still missing to identify those patients with better prognosis, which benefit from intensive systemic treatment or on the contrary patients with very poor prognosis, for which best supportive care could be considered as the best treatment strategy.

Here, we addressed the prognostic value of systemic inflammation, in a predefined systemic inflammation score based on the SIR markers mGPS, NLR, LMR, and CRP in patients with iCCA treated with systemic chemotherapy (Markus et al. 2021). Inflammation is a known hallmark of cancer and promotes tumour growth, invasion, and immunosuppression (Hanahan and Weinberg 2011). Different immune cell types in the tumour microenvironment or in the peripheral blood either mediates pro- or anti-tumoral effects (Hanahan and Weinberg 2011). For example, monocytes, and neutrophils are known suppressors of anti-tumour immunity, whereas lymphocytes as a part of the adaptive immune system can suppress tumour growth (Lin et al. 2019). The composition of the different subtypes of immune cells in the peripheral blood can be easily assessed using the ratios of these leukocyte subsets: NLR and LMR. In addition to the systemic inflammation, the patient’s nutritional status is a strong prognostic factor. The nutritional status could be estimated by the serum albumin levels. Using the composite marker mGPS, the systemic inflammation as well as the nutritional status can be easily assessed in peripheral blood. We included 219 unselected patients with iCCA irrespective of age, comorbidities or performance status treated at our center into this retrospective study. In total, 81 patients were initial resected and 54 of these resected patients had a relapse. Systemic palliative chemotherapy was administered to 119 patients; the remaining patients received only active symptom control, died shortly after initial diagnosis or after relapse. Compared to the pivotal ABC-02 trial or the recently presented TOPAZ-1 trial, the ORR and the PFS upon first-line chemotherapy were markedly lower in our unselected “real-world” patient cohort (Oh et al. 2022; Valle et al. 2010). However, the median OS in the palliative setting was comparable. This difference could be explained by the relative high number of patients, which received second—and further line therapies and the exclusion of patients with extrahepatic BTC and gall bladder cancer. In line, in a pooled post hoc analysis of the ABC-01, -02, and -03 trials, the median OS in patients with iCCA treated with gemcitabine and cisplatin was markedly higher with 15.4 months (Lamarca et al. 2020). All our analysed SIR markers had a negative prognostic impact and correlated with reduced OS in our patients with iCCA treated with systemic chemotherapy. Using our previous established composite systemic inflammation score (SIS), we could assign our patients into three different risk groups and identified patients with low, intermediate, and high risk. Patients in the low-risk group (SIS = 0 and 1) had a remarkable median OS of 28.2 months, whereas patients in the high-risk group (SIS = 3 and 4) had a dramatic short median OS of only 6 months. In line, DCR and PFS to first-line therapy was significantly lower in the high-risk group with a median PFS of only 1.8 months and a high rate of primary progresses at first staging (60%). Interestingly, our proposed SIS was the only independent prognostic factor in multivariate analyses including ECOG, CA 19-9, LDH, and N- and M-status.

Limitations of our study include the retrospective, single-center cohort with possible center and selection bias. From the initially sizeable cohort, for only a limited number of patients, all SIR makers were available. Further validation of our findings in other cohorts or ideally in a prospective study will be necessary.

In conclusion, we identified the SIS as an independent prognostic risk score in patients with advanced iCCA treated with systemic chemotherapy. The SIS allows the assignment of patients into low- and high-risk groups which could be a support for therapeutic decision or a future stratification factor for clinical trials.

## Supplementary Information

Below is the link to the electronic supplementary material.

Supplementary file1 (PDF 18 KB)

Supplementary file2 (PDF 17 KB)

Supplementary file3 (PDF 69 KB)

Supplementary file4 (DOCX 18 KB)

## Data Availability

The data that support the findings of this study are not publicly available due to their containing information that could compromise the privacy of research participants, but are available in processed and anonymized form from the corresponding author.
