# Supplementary figures and images for: A prognostic systemic inflammation score (SIS) in patients with advanced intrahepatic cholangiocarcinoma
Source: J Cancer Res Clin Oncol. 2022 Nov 5;149(8):5085–94. doi: 10.1007/s00432-022-04424-0 (PMC10349723; doi:10.1007/s00432-022-04424-0)

# Overall Survival Upon First Palliative Treatment

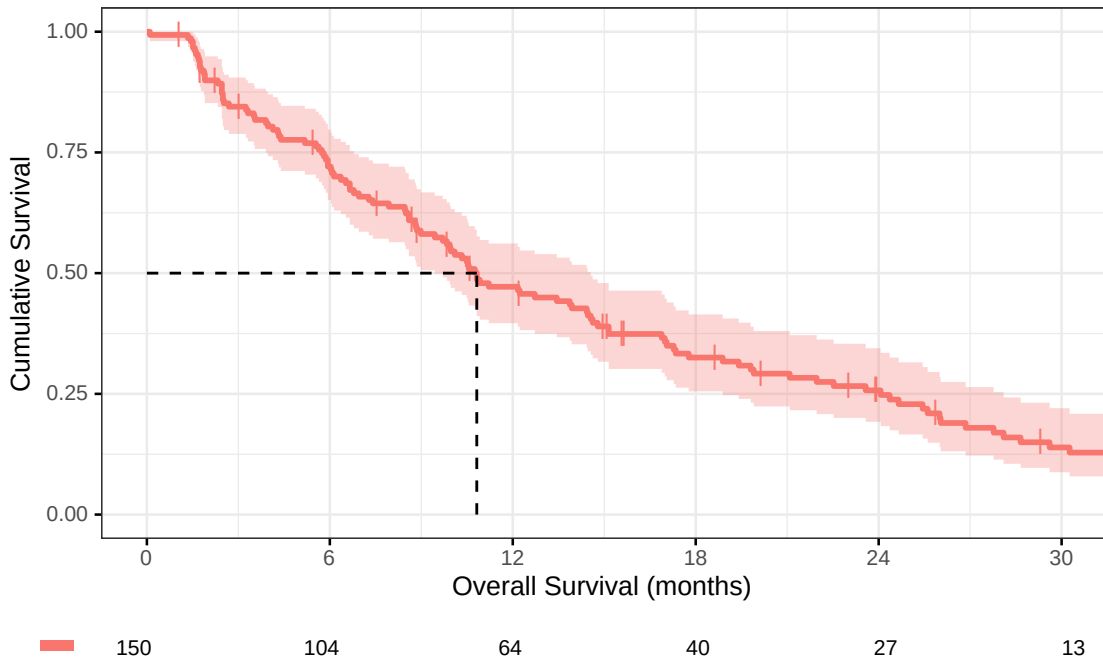

Supplement: Supplementary file 1 — Supplementary file1 (PDF 18 KB) [file 432_2022_4424_MOESM1_ESM.pdf]

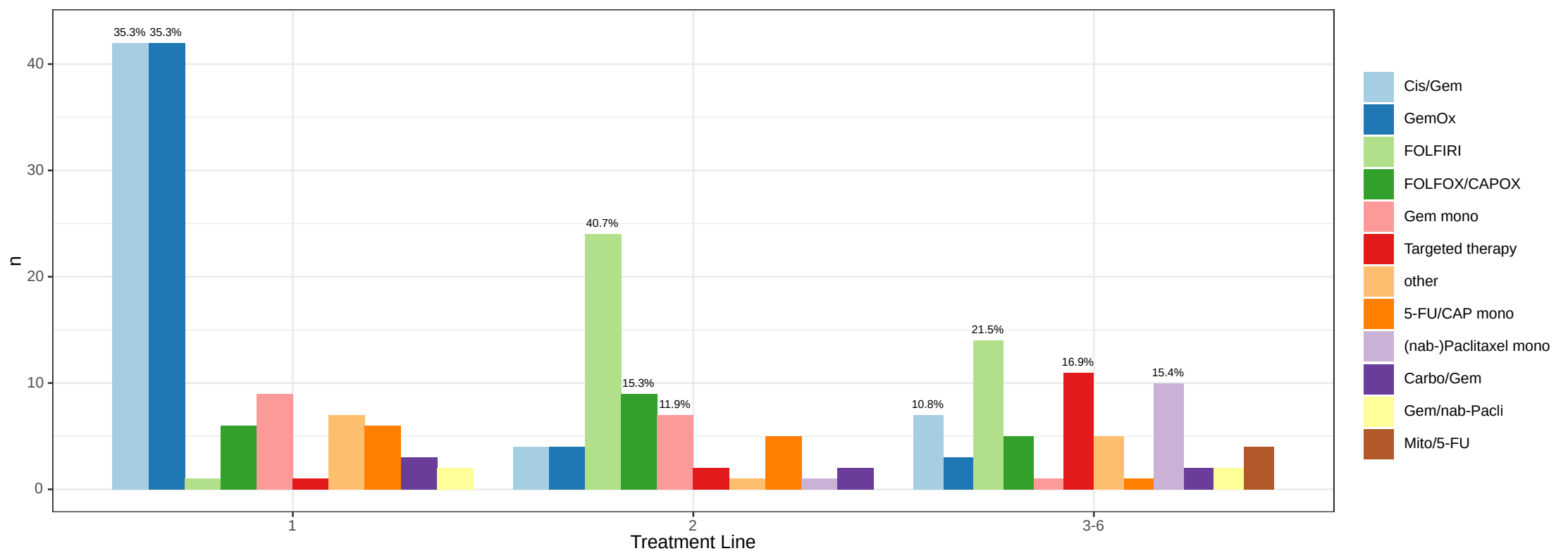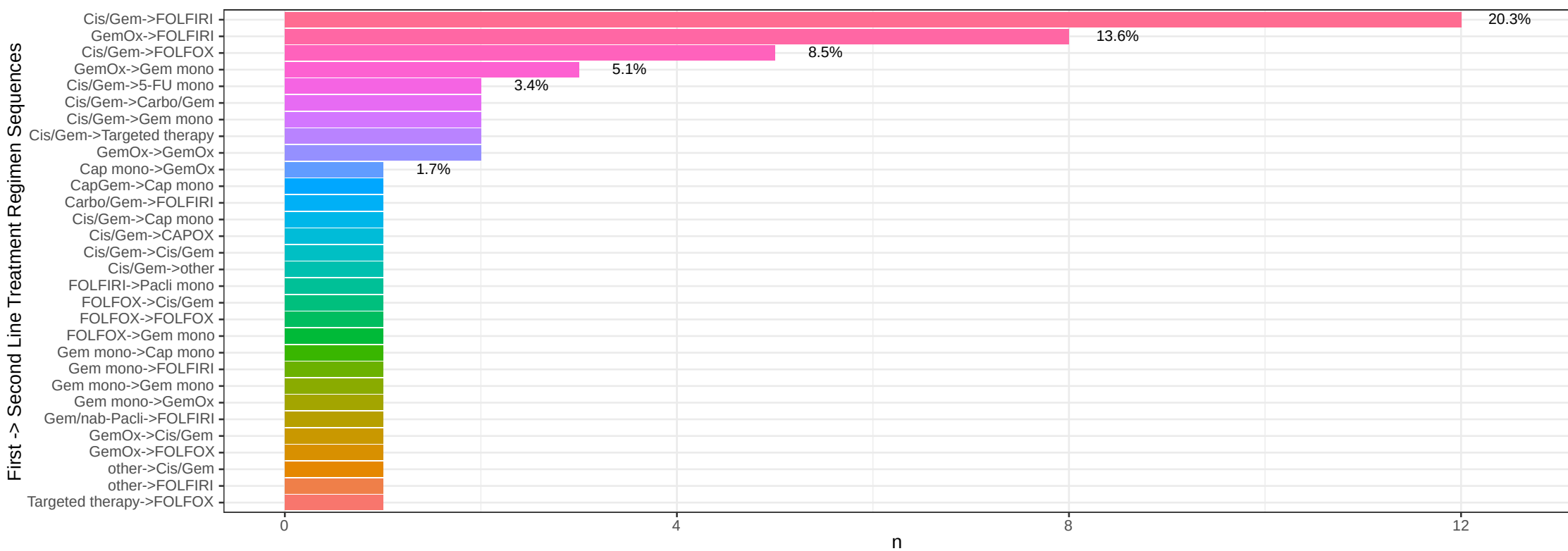

Supplement: Supplementary file 2 — Supplementary file2 (PDF 17 KB) [file 432_2022_4424_MOESM2_ESM.pdf]

# CONSORT diagram

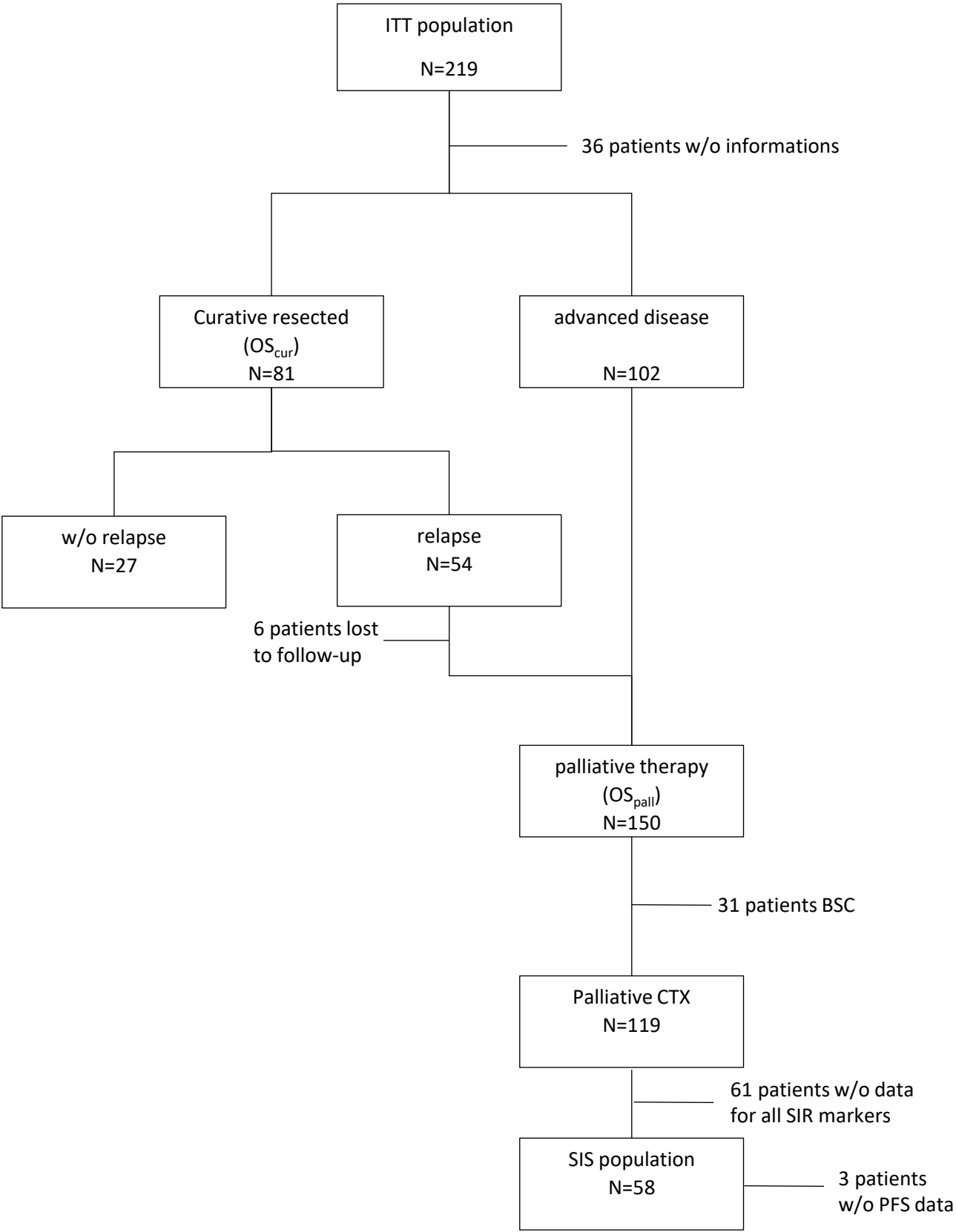

Supplement: Supplementary file 3 — Supplementary file3 (PDF 69 KB) [file 432_2022_4424_MOESM3_ESM.pdf]
