# Supplementary material for: A prognostic systemic inflammation score (SIS) in patients with advanced intrahepatic cholangiocarcinoma
Source: J Cancer Res Clin Oncol. 2022 Nov 5;149(8):5085–94. doi: 10.1007/s00432-022-04424-0 (PMC10349723; doi:10.1007/s00432-022-04424-0)
Supplement: Supplementary file 4 — Supplementary file4 (DOCX 18 KB) [file 432_2022_4424_MOESM4_ESM.docx]

**Supplemental tables and figures**

**Suppl. Table 1- Therapy**

|  | N | % |
| --- | --- | --- |
| Therapy category  Curative intent  Initially palliative  n.d. | 81  102  36 | 37%  47%  16% |
| CTx-lines palliative treatment  1  2  3  4 | 119  59  34  16 | 100%  49.6%  28.6%  13.5% |
|  | | |
| First line (n=119) | | |
| Cisplatin + Gemcitabin | 42 | 35.3% |
| Oxaliplatin + Gemcitabin | 42 | 35.3% |
| Gemcitabin mono | 9 | 7.6% |
|  | | |
| Second line (n=59) | | |
| FOLFIRI | 24 | 40.7% |
| FOLFOX/ CAPOX | 9 | 15.3% |
| Gemcitabin mono | 7 | 11.9% |
|  | | |
| Third and further lines (n=65) | | |
| FOLFIRI | 14 | 21.5% |
| Targeted therapy | 11 | 16.9% |
| (nab)-Paclitaxel mono | 10 | 15.4% |

**Table 1** FOLFIRI, folinic acid, irinotecan, and fluorouracil; FOLFOX, folinic acid, fluorouracil, oxaliplatin

**Suppl. Table 2- Overall survival**

|  | N | Median in months | 95% CI |
| --- | --- | --- | --- |
| OS | 219 | 14.8 | 11.2-24.4 |
| OS_pall_ | 150 | 10.8 | 9.4-14.6 |
| OS_cur_ | 81 | 38.6 | 35.4-47.6 |
| DFS | 81 | 12.3 | 9.7-23.1 |
| PFS | 119 | 3.5 | 2.5-5.5 |
| Efficacy of palliative treatment according to RECIST 1.1 | | | |
| Treatment Line | N | % | 95% CI |
| 1^st^ line  PR  SD  PD  N.e.  ORR (CR+ PR)  DCR (CR+ PR+ SD) | 11  47  33  28  11  58 | 9.2%  39.5%  27.7%  23.5%  9.2%  48.7% | 0.05-0.16  0.31-0.49  0.20-0.37  0.16-0.32 |
| 2^nd^ line  PR  SD  PD  N.e.  ORR (CR+ PR)  DCR (CR+ PR+ SD) | 1  21  23  14  1  22 | 1.7%  35.6%  39.0%  23.7%  1.7%  37.3% | 0.001-0.11  0.24-0.49  0.27-0.53  0.14-0.37 |

**Table 2** OS, Overall Survival; DFS, Disease-free survival; PFS, Progression-free Survival; RECIST, Response evaluation criteria in solid tumours; ORR, Objective Response Rate; DCR, Disease Control Rate; PR, Partial Response; SD, Stable Disease; PD, Progressive Disease; N.e., not evaluable.

| SIS | N | Median OS in months (95% CI) | HR (p-value) |
| --- | --- | --- | --- |
| Low (0-1) | 22 | 28.2 (24.7-NA) | reference |
| Intermediate (2) | 17 | 14.6 (9.9-44.7) | 1.64 (95% CI 0.77-3.47 p 0.196) |
| High (3-4) | 19 | 6.0 (3.2-11.2) | 8.7 (95% CI 3.71-20.38 p <0.001) |

**Suppl. Table 3- Correlation of SIS with OS grouped**

**Table 3** SIS, Systemic Inflammation Score; OS, Overall Survival; CI, Confidence interval; HR, Hazard ratio

**Suppl. Table 4- Correlation of SIS with PFS grouped**

| SIS | N PFS | Median PFS in months (95% CI) | HR (p-value) |
| --- | --- | --- | --- |
| Low (0-1) | 20 | 8.1 (3.5-NA) | reference |
| Intermediate (2) | 16 | 6.7 (2.2-11.4) | 1.45 (95% CI 0.7-3.03 p 0.319) |
| High (3-4) | 19 | 1.8 (1.1-7.8) | 3.56 (95% CI 1.65-7.65 p 0.001) |

**Table 4** SIS, Systemic Inflammation Score; PFS, Progression free survival; CI, Confidence interval; HR, Hazard ratio

**Supplemental figures:**

**Suppl. Figure 1** Overall survival upon first palliative treatment

**Suppl. Figure 2** Chemotherapy regimen in first, second and third- six treatment line. GemOx, gemcitabin and oxaliplatin; FOLFIRI, folinic acid, irinotecan, and fluorouracil; FOLFOX, folinic acid, fluorouracil, oxaliplatin; CAPOX, capecitabin and oxaliplatin; Carbo/Gem, Carboplatin and Gemcitabin; Mito/ 5-FU, mitomycin and fluorouracil

***Suppl. Figure 3*** *CONSORT Diagram. ITT, intention to treat; BSC, best supportive care; SIS, systemic inflammation score; SIR marker, systemic inflammatory response marker; PFS, progression-free survival*
